# Melatonin and Atopy: Role in Atopic Dermatitis and Asthma

**DOI:** 10.3390/ijms150813482

**Published:** 2014-08-04

**Authors:** Lucia Marseglia, Gabriella D’Angelo, Sara Manti, Carmelo Salpietro, Teresa Arrigo, Ignazio Barberi, Russel J. Reiter, Eloisa Gitto

**Affiliations:** 1 Neonatal and Pediatric Intensive Care Unit, Department of Pediatrics, University of Messina, Via Consolare Valeria, 1, 98125 Messina, Italy; E-Mails: gabridangelo@alice.it (G.D.); barberii@unime.it (I.B.); egitto@unime.it (E.G.); 2 Unit of Paediatric Genetics and Immunology, Department of Paediatrics, University of Messina, Via Consolare Valeria, 1, 98125 Messina, Italy; E-Mails: saramanti@hotmail.it (S.M.); carmelo.salpietro@unime.it (C.S.); tarrigo@unime.it (T.A.); 3 Department of Cellular and Structural Biology, University of Texas Health Science Center at San Antonio, San Antonio, TX 40729, USA; E-Mail: reiter@uthscsa.edu

**Keywords:** melatonin, allergy, bronchial asthma, atopic eczema

## Abstract

Melatonin may have important immunostimulatory actions in allergic diseases, in addition to its well-known antioxidant and cytoprotective effects in several inflammatory conditions. The activation of the immune system leads to free radical production associated with decreased melatonin levels and depressed antioxidant enzyme activities in several inflammatory diseases. Many skin disorders, including atopic dermatitis, are accompanied by infiltration and activation of mast cells, which release vasoactive and proinflammatory mediators. Experimental data suggest that melatonin inhibits development of atopic eczema and reduces serum total IgE and IL-4. Allergic asthma is a condition characterized by bronchial hyperresponsiveness and the presence of IgE antibodies in response to inhaled allergens; often there is also enhanced total serum IgE levels. Melatonin regulates smooth muscle tone and influences the immune response. Melatonin may, however, act as a pro-inflammatory agent in asthma leading to bronchial constriction. The safety of melatonin as a sleep-inducing agent has been confirmed in asthmatic subjects, but its routine use is not recommended in bronchial asthma. This review summarizes what is known about the role of melatonin as an immunomodulatory agent in asthma and atopic eczema.

## 1. Introduction

Atopy is an individual or familial tendency to produce IgE antibodies in response to low doses of allergens and to develop typical symptoms such as eczema/dermatitis, rhinoconjunctivitis, or asthma [1]. The development of an immune response depends on a repertoire of cytokines produced by numerous cells, mainly CD4+ helper T cells. These lymphocytes can be paradigmatically divided into two subsets: T helper type 1 (Th-1) and T helper type 2 (Th-2) cells [2]. Effector Th1 cells generate IFN-ɣ and interleukin (IL)-2, whereas molecules produced by Th2 cells are IL-4, IL-9, IL-10 and IL-13 [3]. In atopic subjects, naïve CD4+ T cells differentiate into prevailing Th2 effectors. Furthermore, the cytokines induce the recruitment of pro-inflammatory cells [4]. To date, in addition to Th2/Th1 cells, other T sub-populations such as regulatory T (T-reg) cells and Th17 cells, a novel CD4+ T cell subset, are known to play roles in atopic diseases [5]. In fact, a lower level of T-reg cells has been reported in patients with atopic diseases *versus* healthy controls. Generally, T-reg cells, in addition to T-helper and B-cells, are involved in the release of anti-inflammatory cytokines such as IL-10 [6]. Th17 cells also promote pro-inflammatory cytokine synthesis. Moreover, in response to immune triggering factors, Th17 cells, influenced by IL-23, enhanced cell recruitment and antigen-induced Th2 cytokine release [7]. Although there have been several studies concerning the cytokine profile involved in the phenotypic expression of atopic diseases, their pathogenesis remains unclear.

Oxidative stress stimulates inflammatory responses that can lead to allergic disorders such as atopic dermatitis, allergic rhinitis, and asthma. Melatonin (*N*-acetyl-5-methoxytryptamine) is an indolamine mainly produced in the pineal gland [8]. Extrapineal melatonin synthesis has, to date, been identified in many other site including brain, retina, Harderian gland, ciliary body, lens, thymus, airway epithelium, bone marrow, immune cells, gonads, placenta, gastrointestinal tract and skin [9]. Human skin moreover, expresses both melatonin receptors (MT1 and MT2), although MT1 is the predominant receptor found in whole skin and cultured cells [10]. Melatonin, a powerful endogenous free radical scavenger, also functions as a potent anti-inflammatory agent as documented in both *in vivo* and *in vitro* studies [11,12,13,14]. Melatonin stimulates some important antioxidative enzymes such as superoxide dismutase, glutathione peroxidase and glutathione reductase, protecting cell membranes from lipid peroxidation and neutralizing toxic radicals [15,16,17]. Melatonin plays key roles in a variety of important physiological functions including regulation of circadian rhythms for its sleep-inducing activity [18,19], as well as visual, reproductive, cerebrovascular, neuroendocrine function and it seems to have important neuroimmunological actions and immunomodulatory effects in allergic diseases [20,21]. Because of its beneficial effects, melatonin has been successfully used in the treatment of cancer, sleep disorders, and ageing [22,23,24,25]; however, melatonin’s potential use in atopic patients has rarely been considered [26].

This review summarizes the role of melatonin, as an important immunomodulatory molecule in allergic disorders, including atopic eczema and asthma. Additionally, we examine the studies in which melatonin has been given to patients with these conditions.

## 2. Melatonin and Atopic Eczema

The currently used term for eczematous hypersensitivity reactions in the skin, is “atopic eczema/dermatitis”; this includes atopic/extrinsic, IgE-associated mechanisms, and non-atopic/intrinsic, non IgE-associated forms of these conditions [27].

Atopic eczema (AE) is a multifactorial and chronic relapsing-remitting inflammatory skin disorder frequently beginning in early childhood [28]. Its prevalence is continuously increasing, affecting 15%–30% of children and 2%–10% of the adult population [29]. Although the pathogenesis of AE is complex and partially known, a variety of pathways may be involved in the phenotypic expression of the disease. The immune-pathogenesis of AE is determined by the impairment of different T helper cells as well as their cytokine secretion profiles [30,31,32,33]. AE is mediated by a Th1/Th2 biphasic inflammatory response that, during initiation and maintenance of tissue injury, directly involves several cytokines. In the acute phase, AE lesions are caused by Th2-dependent cytokines, in particular IL-4, involved in IgE switching; IL-5, attracting eosinophils; and IL-13. In chronic AE lesions there is a switch towards a cytokine derived mainly from Th1 cells, leading to interferon (IFN)-ɣ release [34]. AE is characterized by heterogeneous clinical manifestations such as eczema, erythema, edema, excoriation, scaling, and itching. According to the severity of the disease, sleep disturbances have also been reported, due to stress-related chronic disease and impaired immune response. Unfortunately, the underlying relationship between the cutaneous manifestations and the neuroendocrine response is unknown.

The potential utility of melatonin in the treatment of AE has been hypothesized, as it might protect skin integrity and might help to maintain a functional epidermal barrier and, through a variety of antioxidative activities including attenuation of lipid peroxidation [35] and antiapoptotic effects [36]. Melatonin, therefore, could be a key effector in the responses of the mammalian skin while dysregulation of the cutaneous melatoninergic system might be involved in the pathogenesis of common skin disorders. Melatonin influences IFN-ɣ synthesis, and, although there were no significant data on a link between melatonin and atopic diseases, it has been suggested that IFN-ɣ is related to serum melatonin levels and AE. Generally, IFN-ɣ increases melatonin synthesis [37]. AE patients exhibit a lower IFN-ɣ production [38] potentially leading to less melatonin release which, in turn, might contribute to sleep disturbances and further stress in AE patients [39]. Therefore, the use of melatonin has been proposed for subjects with AE affected by “delayed sleep-phase syndrome”, because of its ability to regulate sleep-phase and to enhance immune function [40]. However, no clinical trial investigated the supplement of melatonin in human patients with AE.

Experimental data also suggested that melatonin, as well as its precursor l-tryptophan, might reduce serum total IgE and IL-4 levels thereby impeding the development of AE. However, this immune effect seems also to suppress IFN-ɣ due to activated CD4+ T cells [41]. In fact, it has been demonstrated that melatonin, via its nuclear specific high affinity-binding sites on both Th1 and Th2 as well as its membrane receptors (MT1), prevents IL-2 production promoting defects in Treg activity [41,42]. Conversely, the nocturnal melatonin peak is associated with a high IFN-ɣ/IL-10 and Th1/Th2 ratio [43]. Early nocturnal sleep induces a switch towards Th1 cytokine production while late sleep promotes a shift to a more pronounced Th2 activity [44]. Both Th1 and Th2 express membrane and nuclear receptors for melatonin; via these receptors the indolamine induces the synthesis of pro-inflammatory Th1-cytokines including IFN-ɣ, IL-2, IL-6, and IL-12 [45,46,47] by lymphocytic and monocytic cell lines [48] and amplifies melatonin receptors [49]. Additionally, melatonin influences the activity of pro-inflammatory cells such as NK cells, T and B lymphocytes, granulocytes, monocytes, and mast cells [50]. The latter, which are also activated by neuronal stimulation, induce synthesis of inflammatory mediators including histamine that increases vascular permeability, cytokines, proteases, prostaglandins, leukotrienes [51] and corticotropin-releasing hormone (CRH), implicated in activation of the hypothalamic—pituitary—adrenal (HPA) axis [52] and in melatonin release. Furthermore, melatonin and its metabolites, through the up regulation of antioxidant enzymes, efficiently neutralizes several free radicals and stabilizes cell membranes [17]. Melatonin also seems to prevent degranulation, infiltration and activation of mast cells in the dermis that normally contribute to skin injury. Additionally, although the role of reactive oxygen species (ROS) has rarely been investigated in atopy, recently it has been reported that AE patients are more prone to damage mediated by oxidizing agents. In fact, stress-related disease promotes free radical release, which mediates lipid peroxidation, cell membrane destruction and molecular damage [53]. Melatonin also prevents lipid peroxidation, because of its diverse free radical scavenging activities [54]. Finally, melatonin may be involved in pathogenesis of AE by acting as neuroendocrine factor and the “indole-aminic theory” has been hypothesized. This theory assumes a link between indole-aminic activity, reduced levels of melatonin production, and biological hypersensitivity to environmental triggers. Moreover, immunological defects in AE cause reduced circulating levels of the indolamine, due to less abundant pineal melatonin availability; plasma melatonin levels were found decreased in patients with AE [55]. Moreover Muñoz-Hoyos * et al.* demonstrated that in the phases of AD outbreaks, patients presented a diurnal reduction in the serum levels of melatonin and β-endorphin, although nocturnal levels were greater for both hormones. They hypothesized that the physiological nocturnal peak of melatonin due to pineal gland production might balance the possible decline of melatonin of extrapineal (immunological or cutaneous) origin during episodes of dermatitis outbreaks [56].

Melatonin could be used to treat sleep disorders related to nocturnal itching and the subsequent scratch response typical of children with eczema. Furthermore, in otherwise healthy children, the poor sleep initiation and frequent and prolonged awakenings [57], have been associated with behavioural deficits (e.g., hyperactivity, aggression, anxiety, *etc.*) [58], and reduced neurocognitive performance (e.g., lower IQ, impaired memory, reduced academic performance and attentional capacity, *etc.*) [58].

Despite the fact that melatonin is a molecule with an excellent biosafety profile and any serious side effects [59] or treatment-related complications with short or long-term melatonin therapy in children and adults have been reported [60], to our knowledge, its use in atopy-related sleep has not been specifically evaluated. Moreover, due to its pleiotropic roles and immune activities, melatonin could be hypothesized as a potentially useful agent in the treatment of AE. Obviously, additional studies are needed to clarify the neuro-immunological-hormonal effects of melatonin for its future use as an AE treatment.

## 3. Melatonin and Asthma

Bronchial asthma (BA), a clinical syndrome characterized by chronic airway inflammation, airway responsiveness, and expiratory airflow limitation, is a common chronic health problem in childhood. Available treatments reduce asthma related morbidity, but do not alter the natural history of the disorder [61]. Prevalence of this disease varies from country to country and region to region worldwide [62]. It is thought to develop through complex interactions between genetic and environmental factors. An imbalance between oxidative stress and antioxidant capacity may play an important role in the development and progression of BA. Asthmatic patients are exposed to additional endogenous oxidative stress; their antioxidant system can be overwhelmed in comparison with that of healthy subjects [63,64]. It is known that a deficiency of antioxidants, due to reduced production or insufficient uptake, may contribute to the pathogenesis of BA [65]. Exogenous sources of oxidants include cigarette smoke and toxic gases, while the main source of endogenous oxidants are inflammatory cells [66,67]. These toxic agents cause direct tissue oxidation, release of additional endogenous oxidants, and inactivation of antioxidant defence mechanisms, which interact with other protective agents such as antiproteases [68]. ROS causes bronchial tissue damage, constriction of smooth muscles, increase in vascular permeability, and bronchoconstriction; these play crucial roles in the pathogenesis of BA exacerbations [69]. The episodes of BA are associated with elevated levels of oxidative stress [70].

Because pulmonary and systemic oxidative stress increases inflammatory responses relevant to BA, therapy with antioxidants such as melatonin has been proposed as an approach to reducing asthma incidence or morbidity. However, data concerning the role of melatonin in BA are controversial, and, to date, it is not clear if it has a positive or negative role. Negative effects are mainly related to the pro-inflammatory role of the indolamnine. It has been reported that patients with nocturnal asthma exhibit circadian variations in airway inflammation. The circadian worsening in nocturnal asthma is accompanied by elevated airway inflammation, increased airway responsiveness, and great airflow limitations at night [71]. Melatonin modulates circadian inflammatory variations in nocturnal asthma, causing augmented peripheral blood mononuclear cell production of selected pro-inflammatory cytokines *in vitro* (IL-1, IL-6, and TNF-α), and altering the inflammatory characteristics of peripheral blood mononuclear cells [72,73]. Moreover, the beneficial effect of pinealectomy on cell migration was tested in an experimental model of allergic airway inflammation [74]; this study documented that melatonin replacement in pinealectomized rats restored airway inflammation [74]. Additionally, through promoting the expression of chemotaxins in lung epithelial cells, melatonin might synergize with pro-inflammatory cytokines to modulate BA airway inflammation [75]. Therefore, the development of specific inhibitors of melatonin might paradoxically benefit patients with asthma [74]. Furthermore, melatonin receptors have been isolated in lung tissue [76] and it has been shown that indolamine increases airway smooth muscle tone [77], with a possible negative effect on the severity of asthma. Melatonin levels have been found to be higher in patients with nocturnal asthma than in patients with non-nocturnal asthma and healthy controls, suggesting the possibility that nocturnal exacerbation of asthma may be due to changes in the level of circulating melatonin [78].

On the other hand, several studies have reported the favourable action of melatonin in BA. Partially in contrast with the aforementioned study [78], serum melatonin levels in patients with BA were found to be significantly lower than those in healthy controls [79]. This incongruity could suggest that the response to melatonin would differ between patients with nocturnal asthma, patients with non-nocturnal asthma, and normal control subjects. However, data related to the level of melatonin in asthmatic patients are still inconclusive.

Melatonin is not only a potent free radical scavenger, but also regulates gene transcription involved in mucus production [80]. Recently, in asthmatic animals, it has been reported that melatonin significantly inhibits mucus production by suppression of gene expression. Therefore, it has been assessed that melatonin could represent an effective therapeutic product for management of chronic inflammatory diseases, including asthma [80].

Gumral *et al.* [70] observed that oxidative stress is increased in an exacerbation period of BA in patients, whereas melatonin values and antioxidant enzymes are reduced. Among others enzymes that catalyse the formation of oxidants, eosinophil peroxidase (EPO) is implicated in the pathogenesis of asthma. It has been reported that melatonin may have an important role in the inhibition of the EPO activity in various tissues during inflammation, with implications in the regulation of sleep, inflammation, and infectious disorders [81].

Nuclear factor-kappa B, an important transcription factor in BA, leads to an increase in the expression of many genes responsible for pro-inflammatory cytokines, chemokines, and adhesion molecules [82]. Melatonin has recently been shown to cause partial inhibition of the expression of nuclear factor-kappa B and to down regulate the inducible isoform of nitric oxide synthase activity in lung tissue, in an experimental model of asthma [83].

Some asthmatic subjects have predominantly neutrophilic airway inflammation and the presence of airway neutrophils is correlated with a worse outcome and with a lower response to inhaled steroids [84]. Melatonin inhibits rolling and infiltration of neutrophils and, due to its antioxidant properties, could counteract the superoxide production by neutrophils [85] related to the pathogenesis of the nocturnal exacerbations. 

Sleep disorders, frequent in patients with asthma and allergic diseases, likely in part because of the presence of nocturnal symptoms, are due to pathophysiologic (e.g., inflammatory mediators), environmental (e.g., poor trigger control in the home), individual (e.g., nonadherence to medications), and family/cultural (e.g., cultural beliefs) factors [86]. Several studies have shown cross-sectional associations between poor sleep quality caused by night-time symptoms, poor asthma control, and quality of life [87,88]. Based on studies that reported a negative effect of melatonin in experimental models of BA and higher melatonin levels in patients with nocturnal asthma, to date, the use of exogenous melatonin as a sleep promoter agent, is not recommended in asthmatic patients. However, to our knowledge, the only human study that investigated the effect of melatonin administration, *in vivo*, in asthmatic adults [89], demonstrated that the supplement of 3 mg oral melatonin in a single dose for 28 days, 2 h before bedtime, improved subjective sleep quality in patients with mild or moderate asthma, without significant changes in pulmonary function, asthma symptoms, and use of relief medications [89]. Thus, further studies examining the long-term effects of melatonin on airway inflammation and bronchial hyperresponsiveness in humans are needed before melatonin can be routinely recommended or discouraged in these subjects.

## 4. Conclusions

Melatonin, a key regulator of circadian rhythm homeostasis in humans, is a direct radical scavenger and an indirect antioxidant that stabilizes cell membranes, which makes them more resistant to oxidative damage [90]. Melatonin also appears to have an important immunomodulatory effect in allergic diseases and might be involved in atopic eczema and chronic obstructive pulmonary diseases such as BA. Plasma melatonin levels are generally lower in patients with atopic dermatitis [55] and in the exacerbation period of patients with BA [70].

Inconsistent results have been reported in relation to the use of melatonin as a modulator in these atopic conditions, and data are clearly incomplete. Only one study investigated the reduction of plasma melatonin levels in adults with AE [55] and even more controversial are studies concerning the action of melatonin in asthmatic subjects. Melatonin has been widely used for decades is some countries and no long term toxicity has been reported, but data evaluating the effects of melatonin administration on clinical symptoms and lung function in asthmatic humans are certainly incomplete. While melatonin use is currently not generally recommended for individuals who suffer with asthma, the single published human study related to this issue [89], revealed that melatonin did not worsen symptoms in moderately asthmatic women who used the indolamine to promote sleep. Thus, the preconception that melatonin is detrimental to asthma patients should not be used as rationale for not testing it as a sleep aid or as a potential treatment for asthma in these individuals.
